# The Human Fecal Endocannabinoidome Mediator Profile Is Mainly Defined by the Fecal Microbiota and Diet

**DOI:** 10.1210/clinem/dgae586

**Published:** 2024-08-22

**Authors:** Sophie Castonguay-Paradis, Lydiane Parent, Gabrielle St-Arnaud, Julie Perron, Élizabeth Dumais, Nicolas Flamand, Frédéric Raymond, Vincenzo Di Marzo, Alain Veilleux

**Affiliations:** Centre Nutrition, Santé et Société (NUTRISS), Institut sur la nutrition et les aliments fonctionnels (INAF), Université Laval, Québec, QC, Canada, G1V 0A6; École de nutrition, Faculté des sciences de l’agriculture et de l’alimentation (FSAA), Université Laval, Québec, QC, Canada, G1V 0A6; Canada Research Excellence Chair in the Microbiome-Endocannabinoidome mediators Axis in Metabolic Health (CERC-MEND), Université Laval, Québec, QC, Canada, G1V 0A6; Centre Nutrition, Santé et Société (NUTRISS), Institut sur la nutrition et les aliments fonctionnels (INAF), Université Laval, Québec, QC, Canada, G1V 0A6; Centre Nutrition, Santé et Société (NUTRISS), Institut sur la nutrition et les aliments fonctionnels (INAF), Université Laval, Québec, QC, Canada, G1V 0A6; École de nutrition, Faculté des sciences de l’agriculture et de l’alimentation (FSAA), Université Laval, Québec, QC, Canada, G1V 0A6; Canada Research Excellence Chair in the Microbiome-Endocannabinoidome mediators Axis in Metabolic Health (CERC-MEND), Université Laval, Québec, QC, Canada, G1V 0A6; Centre Nutrition, Santé et Société (NUTRISS), Institut sur la nutrition et les aliments fonctionnels (INAF), Université Laval, Québec, QC, Canada, G1V 0A6; Canada Research Excellence Chair in the Microbiome-Endocannabinoidome mediators Axis in Metabolic Health (CERC-MEND), Université Laval, Québec, QC, Canada, G1V 0A6; Centre de recherche de l’Institut universitaire de cardiologie et de pneumologie de Québec (IUCPQ), Université Laval, Québec, QC, Canada, G1V 4G5; Canada Research Excellence Chair in the Microbiome-Endocannabinoidome mediators Axis in Metabolic Health (CERC-MEND), Université Laval, Québec, QC, Canada, G1V 0A6; Centre de recherche de l’Institut universitaire de cardiologie et de pneumologie de Québec (IUCPQ), Université Laval, Québec, QC, Canada, G1V 4G5; Département de médecine, Faculté de Médecine, Université Laval, Québec, QC, Canada, G1V 0A6; Centre Nutrition, Santé et Société (NUTRISS), Institut sur la nutrition et les aliments fonctionnels (INAF), Université Laval, Québec, QC, Canada, G1V 0A6; École de nutrition, Faculté des sciences de l’agriculture et de l’alimentation (FSAA), Université Laval, Québec, QC, Canada, G1V 0A6; Canada Research Excellence Chair in the Microbiome-Endocannabinoidome mediators Axis in Metabolic Health (CERC-MEND), Université Laval, Québec, QC, Canada, G1V 0A6; Centre Nutrition, Santé et Société (NUTRISS), Institut sur la nutrition et les aliments fonctionnels (INAF), Université Laval, Québec, QC, Canada, G1V 0A6; École de nutrition, Faculté des sciences de l’agriculture et de l’alimentation (FSAA), Université Laval, Québec, QC, Canada, G1V 0A6; Canada Research Excellence Chair in the Microbiome-Endocannabinoidome mediators Axis in Metabolic Health (CERC-MEND), Université Laval, Québec, QC, Canada, G1V 0A6; Centre de recherche de l’Institut universitaire de cardiologie et de pneumologie de Québec (IUCPQ), Université Laval, Québec, QC, Canada, G1V 4G5; Département de médecine, Faculté de Médecine, Université Laval, Québec, QC, Canada, G1V 0A6; Joint International Unit on Chemical and Biomolecular Research on the Microbiome and its Impact on Metabolic Health and Nutrition (UMI-MicroMeNu), Québec, QC, Canada, G1V 0A6; Centre Nutrition, Santé et Société (NUTRISS), Institut sur la nutrition et les aliments fonctionnels (INAF), Université Laval, Québec, QC, Canada, G1V 0A6; École de nutrition, Faculté des sciences de l’agriculture et de l’alimentation (FSAA), Université Laval, Québec, QC, Canada, G1V 0A6; Canada Research Excellence Chair in the Microbiome-Endocannabinoidome mediators Axis in Metabolic Health (CERC-MEND), Université Laval, Québec, QC, Canada, G1V 0A6

**Keywords:** 2-monoacyl-glycerols, *N*-acyl-ethanolamines, dietary intakes, gut microbiota, feces

## Abstract

**Context:**

The endocannabinoid system and its extension, the endocannabinoidome (eCBome), are involved in numerous biological processes, notably energy homeostasis, across virtually all tissues. While the circulating eCBome mediator profile is associated with dietary intakes and metabolic status, an important knowledge gap resides in the identification of the precise determinants of these mediators in the gut lumen.

**Objective:**

We aimed at establishing the profile of eCBome mediators in human feces and investigating their association with circulating eCBome mediators, dietary intakes, metabolic status, and gut microbiota composition.

**Methods:**

*N*-acyl-ethanolamines (NAEs) and 2-monoacyl-glycerols (2-MAGs) were profiled by liquid chromatography coupled to tandem mass spectrometry in plasma and feces of a cross-sectional cohort (n = 195) and a short-term dietary intervention trial (n = 21) with comprehensive dietary intakes and gut microbiota measures.

**Results:**

Six NAEs and 7 2-MAGs were identified in fecal samples, but some, especially omega-3–derived mediators, were undetectable in the majority of samples. Fecal NAEs, and to a lower extent 2-MAGs, were positively albeit weakly correlated with the circulating levels of eCBome mediators. Fecal 2-arachidonoyl-glycerol, *N*-palmitoyl-ethanolamine, and *N*-docosahexaenoyl-ethanolamine levels were positively associated with visceral adiposity and with some parameters of the metabolic profile. Dietary intakes of foods rich in fibers were associated with lower fecal levels of several eCBome mediators, while intakes of unsaturated fatty acids were associated with fecal 2-oleoyl-glycerol and 2-linoleoyl-glycerol. Interestingly, gut microbiota diversity and composition were a strong correlate of the fecal eCBome profile.

**Conclusion:**

The fecal eCBome profile is associated with gut microbiota composition and dietary intakes, more than with the circulating profile. These results strengthen the hypothesis of an interrelation between the gut microbiome and eCBome signaling involved in the regulation of numerous host biological processes.

The expanded endocannabinoid system, or endocannabinoidome (eCBome), encompasses several signaling lipids, of which the *N-*acyl-ethanolamines (NAEs, like the endocannabinoid *N*-arachidonoyl-ethanolamine [AEA] or anandamide) and the 2-monoacyl-glycerols (2-MAGs, such as the endocannabinoid 2-arachidonoyl-glycerol [2-AG]), involved in, among others, energy homeostasis and inflammation processes, are the most studied (1, 2). The eCBome also includes anabolic and catabolic enzymes for NAEs and 2-MAGs, and their several receptors, such as the cannabinoid receptors (CB_1_ and CB_2_), other G-protein–coupled receptors, ligand-activated ion channels such as the transient receptor potential vanilloid type-1 (TRPV1) channels, and peroxisome proliferator-activated receptors (PPARs) α and γ. The eCBome mediators are found in different levels in virtually all tissues as well as in biological fluids such as blood, saliva, and feces (3-5). Given the different range of receptors that each eCBome mediator can modulate locally, and the different (and often opposing) role that these receptors play in the control of energy homeostasis and intestinal epithelial integrity, investigating the effect of diet on eCBome mediator profiles in tissues and biological fluids is extremely important for both the prevention and the pharmacological and dietary management of metabolic disorders.

We have recently highlighted a bidirectional relationship between the eCBome and the gut microbiota. Indeed, alteration of the eCBome tone is associated with specific changes in the composition of the gut microbiota, and vice versa (1, 6). Moreover, some molecules chemically similar to eCBome mediators have been shown to be produced by gut bacteria (7, 8), which were recently also suggested to respond to NAEs and 2-MAGs in culture (9, 10). We have reported that circulating levels of eCBome mediators are associated with total adiposity, fat distribution, and recent dietary intakes of the corresponding fatty acids (3). On the other hand, very few authors have reported the presence of eCBome mediators in feces. None of these studies have identified the determinants of fecal eCBome mediators, and their methods only targeted a few such molecules (4). It is not clear if and to what extent the amounts of NAEs and 2-MAGs found in feces reflect their production and release from the intestinal epithelium, the gut microbiota, or both, and, hence, whether the diet can modify the fecal content of eCBome mediators through its effect on the host, the gut microbiome, or both. This information is crucial to understand if and how the fecal eCBome is involved in the control of host and gut microbe functions, and their communication, particularly in relation to metabolic and intestinal function homeostasis.

We hypothesized that the fecal eCBome mediator profile may mirror the circulating profile and that its determinants, especially the dietary intake of fatty acids, are therefore similar to those of the circulating mediators. Considering its metabolic activity and varied response to diet, we also hypothesized that the gut microbiota and its composition may influence the profile of fecal eCBome mediators. Thus, in this study we aimed at quantifying a large array of NAE and 2-MAG eCBome mediators in the feces of healthy individuals, and identifying the host, gut bacterial, and dietary determinants of these mediators in the feces.

## Materials and Methods

### Study Cohorts

The cross-sectional study sample (eMECA, NCT03463304) includes healthy women (n = 102) and men (n = 93) covering a large range of adiposity phenotypes (Table 1), as previously described (3). Participants included individuals with previous diagnoses of type 2 diabetes (n = 5), hypertension (n = 21), and dyslipidemia (n = 21). Briefly, overnight fasting blood samples were drawn at INAF's Clinical Investigation Unit (Québec City, Canada), and feces samples were collected by the participants using standardized procedures during the day preceding the blood sampling. Detailed data collecting and sampling procedures have been previously described (3). The controlled feeding intervention group (META, NCT03783260) includes 21 healthy men (n = 10) and women (n = 11) with a normal weight (Table 2) in a fixed sequence isocaloric feeding study with a run-in control diet (13 days) followed with a Mediterranean diet (2 days). The control diet was designed to reflect current Canadian macronutrient intakes with low intakes of fiber and high intakes of saturated fat. The Mediterranean diet was characterized by greater intake of fruit and vegetables, plant-based proteins, whole grains, oleic acid, and omega-3 fatty acids (11, 12). Written informed consent was obtained and the studies were approved by the Laval University Ethics Committee (2017-328 and 2018-262).

**Table 1.** Characteristics of the cross-sectional study sample, n = 195

	Range	Mean (SD)
Age (year)	19-85	41 (17.5)
BMI (kg/m^2^)	13.3-42.0	25.0 (4.7)
Fat mass (kg)	4.7-54	22.0 (10.0)
Visceral fat mass (kg)	0-4.3	0.7 (0.8)
Fasting glucose (mmol/L)	3.7-10.2	5.0 (0.7)
Fasting insulin (pmol/L)	9.0-188	45.6 (30.4)
HbA1c (%)	4.3-8.2	5.2 (0.4)
Triglycerides (mmol/L)	0.4-4.5	1.1 (0.6)
Total cholesterol (mmol/L)	1.8-7.7	4.6 (1.1)
HDL cholesterol (mmol/L)	0.6-3.3	1.6 (0.5)
LDL cholesterol (mmol/L)	0.6-5.5	2.5 (0.9)

Abbreviations: HbA1c, glycated hemoglobin; HDL, high-density lipoprotein; LDL, low-density lipoprotein.

**Table 2.** Characteristic of the intervention sample, n = 21

	Range	Mean (SD)
Age (year)	20-34	25 (4.2)
BMI (kg/m^2^)	20.1-25	21.9 (1.4)
Waist circumference (cm)	65.8-87.2	74.9 (5.3)
Fasting glucose (mmol/L)	4.0-4.9	4.5 (0.3)
Fasting insulin (pmol/L)	15-74	40 (18)
Triglycerides (mmol/L)	0.61-1.8	0.8 (0.3)
Total cholesterol (mmol/L)	2.5-5	4.1 (0.7)
HDL cholesterol (mmol/L)	1.1-2.3	1.7 (0.3)
LDL cholesterol (mmol/L)	0.9-3.0	2.1 (0.6)

Abbreviations: BMI, body mass index; HDL, high-density lipoprotein; LDL, low-density lipoprotein.

### Dietary Assessment

Dietary intakes of the cross-sectional sample were assessed using a validated web-based 24-hour dietary recall (R24W) (13). Two R24Ws were spread out over the course of the week preceding sampling, and the third R24W was completed by the participant the day preceding blood and fecal sample collection. The mean of the 3 24-hour dietary recalls was used in the analysis.

### Circulating eCBome Mediator Profiling

NAE and 2-MAG mediators were measured in fecal samples (10 mg) and plasma (200 µL) using high-performance liquid chromatography coupled to tandem mass spectrometry, as previously described (14). The method allows the quantification of NAEs, including AEA, *N*-palmitoyl-ethanolamine (PEA), *N*-oleoyl-ethanolamine (OEA), *N*-linoleoyl-ethanolamine (LEA), *N*-docosapentaenoyl-ethanolamine, *N*-eicosapentaenoyl-ethanolamine (EPEA), and *N*-docosahexaenoyl-ethanolamine (DHEA), as well as 2-MAGs, including 2-AG, 2-palmitoyl-glycerol (2-PG), 2-oleoyl-glycerol (2-OG), 2-linoleoyl-glycerol (2-LG), 2-eicosapentaenoyl-glycerol (2-EPG), 2-docosapentaenoyl-glycerol (2-DPG), and 2-docosahexaenoyl-glycerol (2-DHG). MAG isomers at positions 1 and 2 can be differentiated, but due to their rapid interconversion and the stereospecificity of polyunsaturated fatty acid (PUFA) and monounsaturated fatty acid phospholipid esterification, *sn*-1(3) and *sn*-2 MAGs derived from unsaturated fatty acids were summed up and reported as 2-MAGs. For correlations and multiple factor analysis (MFA), fecal eCBome mediators below the limit of detection (LOD) were imputed between 50% and 100% of the LOD for the corresponding congeners using a beta function. The imputation method was selected based on the assumption that missing values were related to the detection limit (missing not at random). This approach effectively mitigated the impact of low or undetectable eCBome mediator levels without altering the relationships between variables. It is probable that some of 0 values correspond to a complete absence of the mediators, but this remains less likely considering the ubiquitous nature of these molecules and the imputation method does not infringe upon this hypothesis. All analyses were initially conducted using raw data to ensure that imputation did not affect the relationships between variables before proceeding with subsequent analyses.

### 16S rRNA Gene Sequencing

Stool bacterial DNA was extracted using the QIAamp DNA Stool Kit (QIAGEN, CA, USA) and amplification of the V3-V4 region was performed using the primers 341F and 805R (Illumina, CA, USA), as previously described (15). Libraries were purified using magnetic beads (Axygen Biosciences, CA, USA) and high-throughput sequencing (2 × 300 bp paired end) was performed on a MiSeq (15). Sequences were processed using the Dada2 package (Version 1.10.1) (16) and associations to bacterial taxa were obtained using the Silva v132 reference database (17). Sequences present in fewer than 5 samples were filtered out and bacterial abundances were normalized using cumulative sum scaling (MetagenomeSeq R package). Analyses were mainly performed using the families, since preliminary analysis reveals that this taxonomic level explains a large part of the gut microbiota variance, provides stronger statistical power, and reduces the incidence of 0 values compared with genera. Sequencing data for the 16S rRNA sequences were deposited in the NCBI GenBank under BioProject ID PRJNA644138 and under SRA accession number SUB7687442.

### Statistical Analyses

All statistical analyses were conducted with R software version 4.1.1. The Spearman correlation coefficients were computed using *ppcor* R package (18). Ward's hierarchical agglomerative clustering with Euclidian distance (hclust, *stats* R Package) and MFA (*FactoMineR* R package) was used to stratify fecal eCBome profiles and to compare clusters, respectively (19, 20). The MFA model was computed using the relative abundance of all gut microbiota taxonomic levels. One-way analysis of variance and Tukey HSD post hoc test were performed to compare parameters between tertiles or clusters. Paired t-tests were used to compare the control diet and the Mediterranean diet intervention.

## Results

### Fecal eCBome Mediator Profile

**Figure 1.**
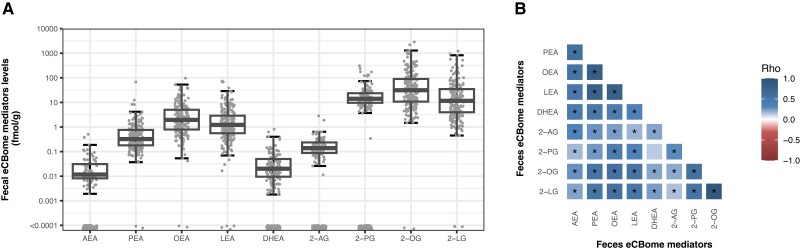
Interrelation of the fecal and the circulating eCBome mediator profile. (A) Boxplot of fecal eCBome mediator levels of NAEs and 2-MAGs. (B) Heatmap representation of Pearson's correlation coefficients between eCBome mediators in the feces (n = 195). *Significant correlation coefficient (*P* < .05).

Quantification of NAE and 2-MAG congeners revealed that all NAEs and 2-MAGs were detectable in feces. Nevertheless, as highlighted in Table 3 and Fig. 1A, the levels of fecal eCBome mediators showed high interindividual variability and some mediators were not observed in all individuals. Indeed, fecal levels of PEA, OEA, LEA, 2-OG, and 2-LG were relatively high and above the LOD for nearly all fecal samples, while only half of the fecal samples harbored detectable levels of AEA, DHEA, 2-AG, and 2-PG (Table 3 and Fig. 1A). Of note, most omega-3–derived eCBome mediators (ie, EPEA, 2-EPG, 2-DPG, and 2-DHG) were only observed in a limited number of fecal samples (Table 3).

**Table 3.** Endocannabinoidome mediator levels in fecal samples, n = 195

	Sample < LOD(%)	Entire cohort		Men, n = 93	Women, n = 102
		Min–Max(pmol/mg)	Mean (SD) (pmol/mg)	Mean (SD)(pmol/mg)	Mean (SD)(pmol/mg)
**NAEs**					
AEA	59	0.002-0.50	0.04 (0.07)	0.05 (0.08)	0.03 (0.06)
PEA	0	0.04-68.2	1.11 (5.04)	1.60 (7.14)	0.65 (1.40)*^a^*
OEA	0	0.04-96.7	5.02 (9.93)	5.47 (9.60)	4.61 (10.3)
LEA	1	0.02-73.6	4.05 (9.78)	4.68 (9.28)	3.50 (10.2)
EPEA	90	0.0007-0.08	0.01 (0.02)	0.005 (0.006)	0.02 (0.03)
DHEA	35	0.002-0.82	0.05 (0.11)	0.06 (0.13)	0.04 (0.07)
**2-MAGs**					
2-AG	50	0.01-2.84	0.23 (0.35)	0.27 (0.44)	0.17 (0.12)*^a^*
2-PG	43	3.7-310	28.9 (46.5)	31.9 (41.2)	25.8 (50.6)
2-OG	0	1.5-2873	125 (314)	165 (331)	89.5 (294)*^a^*
2-LG	1	0.5-1216	54 (147)	78.5 (198)	31 (65.7)
2-EPG	94	0.04-10.6	1.5 (3.5)	0.53 (0.43)	2.28 (4.67)
2-DPG	84	0.01-0.59	0.10 (0.14)	0.10 (0.09)	0.10 (0.17)
2-DHG	86	0.02-17.4	0.87 (3.45)	0.24 (0.37)	1.82 (5.46)

Abbreviations: 2-AG, 2-arachidonoyl-glycerol; 2-DHG, 2-docosahexaenoyl-glycerol; 2-DPG, 2-docosapentaenoyl-glycerol; 2-EPG, 2-eicosapentaenoyl-glycerol; 2-LG, 2-linoleoyl-glycerol; 2-MAG, 2-mono-acyl-glycerol; 2-OG, 2-oleoyl-glycerol; 2-PG, 2- palmitoyl-glycerol; AEA, *N*-arachidonoyl-ethanolamine; DHEA, *N*-docosahexaenoyl-ethanolamine; EPEA, *N*-eicosapentaenoyl-ethanolamine; LEA, *N*-linoleoyl-ethanolamine; NAE, N-acyl-ethanolamine; OEA, *N*-oleoyl-ethanolamine; PEA, *N*-palmitoyl-ethanolamine.

^*a*^Significant differences between men and women. Associations between mediators and sex were not significant once adjusted for adiposity differences.

**Figure 2.**
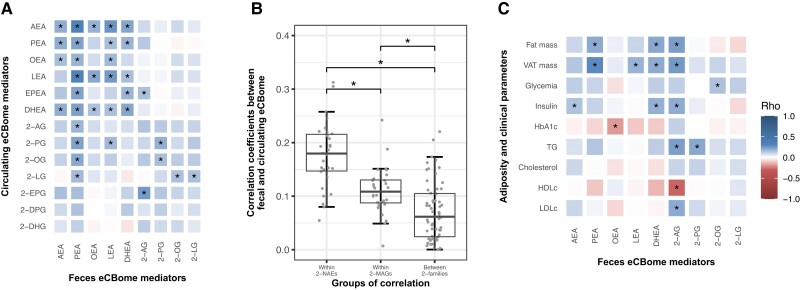
Fecal eCBome association with metabolic status and dietary intakes. (A) Heatmap representation of Pearson's correlation coefficients between circulating and feces eCBome mediators. (B) Boxplot of Pearson's correlation coefficient within NAE mediators and 2-MAG mediators or between NAEs and 2-MAGs in the feces and the circulation. (C) Heatmap representation of Spearman's correlation coefficients of fecal eCBome mediators with adiposity measures and biochemical parameters (n = 195). *Indicates significant correlations coefficient (*P* < .05).

Abbreviations: BMI, body mass index; HbA1c, glycated hemoglobin; TG, triglycerides; HDLc, high-density lipoprotein cholesterol; LDLc, low-density lipoprotein cholesterol.

NAE and 2-MAG congeners were positively intercorrelated in the feces (Fig. 1B). The levels of eCBome mediators in the feces were also generally positively associated with the levels of eCBome mediators in the circulation (Fig. 2A). These correlations were, however, of lower strength than those observed within the feces. Interestingly, feces to plasma correlations within NAEs were, on average, stronger than correlations within 2-MAGs or between NAEs and 2-MAGs (Fig. 2B). These results suggest that the fecal eCBome profile, and particularly that defined by 2-MAGs, is largely independent from the circulating eCBome profile.

### Fecal eCBome, Metabolic Status and Dietary Intakes

We previously reported a tight association between the circulating eCBome profile with adiposity and the metabolic profile (3, 21). Here, we found associations between individual fecal eCBome mediators and adiposity and metabolic parameters, but these associations were not as consistent across fecal NAE and 2-MAG congeners as for the circulating levels in the same individuals (Fig. 2C). Indeed, only fecal PEA, DHEA, and 2-AG levels were positively and significantly associated with total and visceral adiposity. The levels of 2-AG were also associated with higher fasting insulin and triglycerides. Interestingly, fecal 2-AG levels were also associated with higher low-density lipoprotein cholesterol, but lower high-density lipoprotein cholesterol. Interestingly, fecal levels of PEA, 2-AG, and 2-OG were significantly higher in men than in women (Table 3). These associations were not significant once adjusted for adiposity, suggesting that sex differences in adiposity drive these associations.

**Figure 3.**
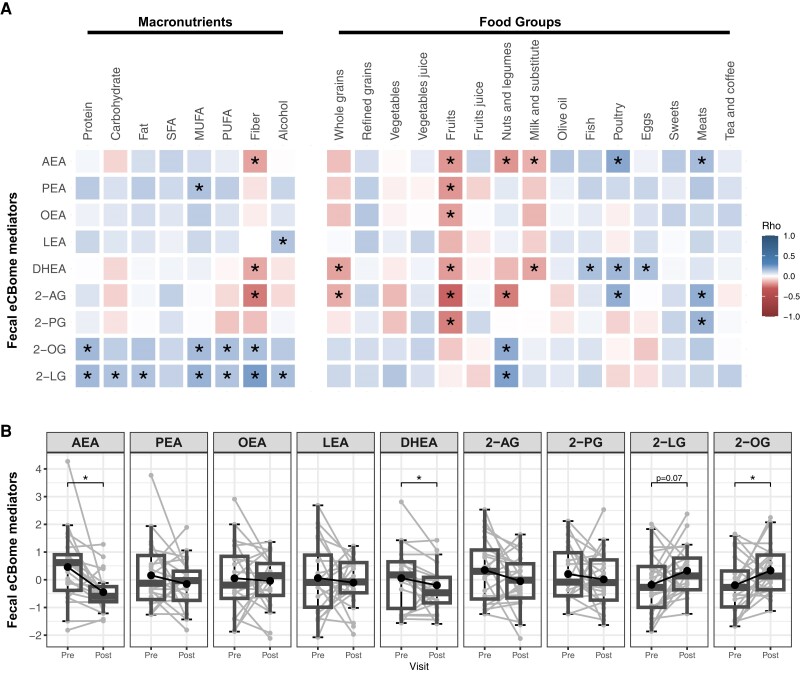
Fecal eCBome is modulated by recent dietary intakes. (A) Heatmap representation of Spearman's correlation coefficient of fecal eCBome mediators with macronutrients and food groups intakes (n = 195). *Statistically significant (*P* < .05). (B) Fecal eCBome mediator levels at the end of the 13-day run-in control diet (Pre) and immediately following the 2-day dietary intervention with high-quality meals reflecting the traditional Mediterranean diet (Post, n = 21). *Statistically significant difference between pre-and postintervention (paired t-test, *P* < .05).

Fecal eCBome associations with dietary intakes, both as macronutrients and food groups, are represented in Fig. 3A. The fiber intake was negatively correlated with fecal AEA, DHEA, and 2-AG levels and positively correlated with fecal 2-OG and 2-LG levels. Interestingly, 2-LG and, to a lesser extent, 2-OG showed a positive association with the intake of all macronutrients. In contrast to what was observed in the circulation, eCBome mediators in the feces were not consistently associated with dietary intakes of their corresponding fatty acid precursors. Indeed, only 2-OG and 2-LG were positively associated with both monounsaturated and polyunsaturated fat intakes. In addition, the fecal eCBome profile was associated with dietary intakes reported as food groups. AEA and 2-AG were both positively associated with intakes of poultry and meats. Fruit consumption was negatively associated with fecal levels of NAEs, as well as of 2-AG and 2-PG. Interestingly, fish consumption was positively associated with fecal DHEA levels. Nut and legume consumption was positively associated with fecal 2-OG and 2-LG levels, and negatively associated with fecal AEA and 2-AG levels (Fig. 3A).

### Fecal eCBome Mediator Short-term Response to a High-Quality Dietary Intervention

Considering the association between the fecal eCBome profile and dietary intakes, we tested whether the fecal eCBome mediators were modulated by the transition from a relatively low-quality run-in diet to a high-quality diet (META trial (12)). The results revealed that the 2-day dietary intervention, notably rich in fibers, unsaturated fatty acids, and plant-based proteins, significantly altered the levels of some, but not all, eCBome mediators in the feces (Fig. 3B). Notably, 2-LG and, particularly, 2-OG, were increased following the 2-day high-quality diet intervention in accordance with the higher intake of oleic and linoleic acid, respectively. Fecal levels of AEA and DHEA were decreased following the high-quality diet intervention (Fig. 3B). All other eCBome mediators remained unaffected by the dietary intervention even though the dietary intervention profoundly modified the intakes of their precursor fatty acids.

### Fecal eCBome and Gut Microbiota Composition

**Figure 4.**
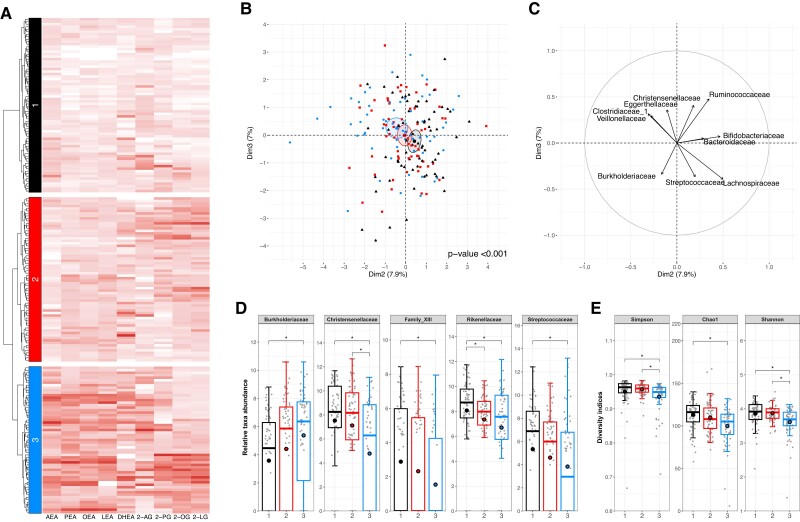
Fecal eCBome profiles cluster with gut microbiota diversity and composition. (A) Dendrogram and heatmap representation of Ward's (20) hierarchical agglomerative clustering of fecal eCBome mediators. Heatmap shows normalized fecal eCBome mediator levels of each s by individuals according to the clusters. Higher relative values are shown by a darker fill. (B) Graph of individuals of the multiple factor analysis (MFA) overlaid with the cluster of fecal eCBome mediator profile. The MFA model was computed using the relative abundance of all gut microbiota taxonomic levels. Ellipses represent the standard deviation from the mean center of each group of individuals. The permutational multivariate analysis of variance *P* value for the difference between cluster is included. (C) Loading plot of the MFA model highlighting the contribution of factors at the family taxonomic levels. (D) Boxplot of families and (E) diversity indexes (Shannon, Simpson, and Chao1) of the gut microbiota composition, according to the fecal eCBome mediator clusters. Boxplots include the median, lower/higher quartiles and 1.5*x* interquartile range whiskers. The means of the distribution is represented by a red dot (n = 70, n = 66 and n = 59 for clusters 1, 2, and 3 respectively). *Statistically significant differences between clusters (Pairwise Wilcoxon Rank tests with Holm correction, *P* < .05, n = 195).

Hierarchical clustering analysis identified 3 distinct profiles of fecal eCBome mediators in the cross-sectional cohort of healthy individuals (Fig. 4A). The first cluster was characterized by relatively low levels of all eCBome mediators in the feces. By contrast, individuals in the third cluster had higher fecal levels of all NAE and 2-MAG congeners. The second cluster falls in between, showing intermediate levels of most eCBome mediators, especially for 2-MAGs. A MFA model, including all gut microbiota taxonomic ranks, reveals that the fecal eCBome clusters are associated with change in gut microbiota composition (Fig. 4B). Indeed, the second and third dimension of the model enables the stratification of the 3 profiles of fecal eCBome mediators. In Fig. 4C, the loading plot shows the main contributors of the MFA at the family taxonomic rank. Individuals in the clusters harboring higher fecal eCBome mediator levels had lower relative abundance of the Christensenellaceae, Clostridiales Family XIII, Rikenellaceae, and Streptococcaceae families, but higher relative abundance of the Burkholderiaceae family (Fig. 4D). In addition, the clustering according to the fecal eCBome mediators was associated with gut microbiota diversity, such as individuals with higher fecal eCBome levels were characterized by a lower gut microbiota diversity (Fig. 4E).

## Discussion

Recent evidence supports a bidirectional relationship between the eCBome and the gut microbiome. We previously reported that circulating levels of eCBome mediators are associated with total adiposity, fat distribution, recent dietary intakes, and gut microbiota composition (3, 12). Nevertheless, a knowledge gap persists on which eCBome mediators are in the gut lumen and how they are associated with host systemic context or with commensal bacteria metabolic activity. We thus aimed to profile NAE and 2-MAG eCBome mediators in the feces of healthy individuals to identify the host and gut microbiota determinants of these mediators in the feces. We showed that most NAEs and 2-MAGs are present in human feces, although omega-3–derived eCBome mediators were found at very low levels. Fecal eCBome mediators were strongly correlated within each family and between NAEs and 2-MAGs. However, fecal eCBome mediators were not strongly correlated with circulating eCBome mediators, except, somehow weakly, within NAEs. Indeed the fecal eCBome profile exhibited partial independence from the circulating eCBome profile, as also witnessed by the finding that fecal eCBome mediators showed a distinct association pattern with adiposity, metabolic parameters, and dietary intakes compared with the circulating metabolites (3, 21). We also highlighted an association between fecal eCBome mediators and gut microbiota composition. Indeed, increased levels of eCBome mediators in the feces were associated with reduced gut microbiota diversity and altered taxonomic relative abundance.

To our knowledge, this study is the first to profile a large array of NAE and 2-MAG congeners in human feces. Quantifying these lipid mediators in the fecal matrix remains complex but our extraction and liquid chromatography coupled to tandem mass spectrometry protocols proved effective in quantifying fecal levels of most of the congeners previously found in the plasma. Indeed, we found 2-LG and 2-OG in all fecal samples, while these mediators were detected in less than 40% of the samples by Goedert et al (22). Nevertheless, some of the mediators we have identified with our method remain undetectable in the feces of a large proportion of individuals. Notably, omega-3–derived NAEs and 2-MAGs were only detected in a few samples. At this stage, it is unclear whether these molecules are virtually absent or present in amounts just below the detection limit. Given that nearly all dietary fatty acids are absorbed in the small intestine, and that omega-3 fatty acids constitute only a small fraction of these lipids, we may hypothesize that the pool of luminal omega-3 fatty acids is insufficient to result in significant quantities of these mediators. By contrast, oleic and linolenic acids represent a substantial proportion of the dietary fatty acids, potentially favoring the production of their corresponding eCBome mediators in the gut lumen. Interestingly, the changes in omega-3–derived eCBome mediators following the short, high-quality dietary intervention tend to support this hypothesis. However, the most common omega-3–derived eCBome mediator in feces (ie, DHEA) decreased even when individuals had substantially increased their intakes of its direct precursor (ie, DHA) during the short-term intervention. By contrast, the increased intake of linoleic and, particularly, oleic acids during the 2-day dietary intervention was mirrored in the fecal levels of the corresponding 2-MAG congeners, but not of the NAE congeners. In view of our previous finding of a significant increase in circulating levels of DHEA and OEA following the same dietary intervention (3), these data confirm that the fecal eCBome mediator profile is not determined by the same factors as the host eCBome profile. The fecal eCBome, especially for what concerns NAEs, may also result from the biosynthetic and metabolic activity of the gut microbiota. In this sense, it is possible that (1) lower omega-3-PUFA levels in the gut lumen, (2) inability of commensal bacteria to synthesize omega-3-PUFA derived NAEs, and/or (3) higher catabolic activity of these mediators explain the diverging profile of eCBome mediators in the feces compared with the plasma. This possibility, which is currently being investigated in our laboratory, may have important functional consequences, given the points raised in (1) and below.

Following in the same line of reasoning, the intriguing decrease in DHEA levels following the high-quality diet, regardless of the precursor fatty acid intakes, may be the result of changes in the gut microbiota composition and metabolic activity caused by other components of this fiber-rich, plant-based diet. We found that the gut microbiota diversity and the relative abundance of specific taxa were associated with the levels of fecal eCBome mediator levels. More precisely, higher fecal levels of most NAE and 2-MAG eCBome mediators were associated with a lower diversity and a lower relative abundance of the Christensenellaceae, Clostridiales Family XIII, Rikenellaceae and Streptococcaceae families, but a higher relative abundance of the Burkholderiaceae family. Of these families, variedly associated with obesity, Christensenellaceae were consistently reported to be negatively associated with adiposity and dysmetabolic parameters and to play a potential protective function against obesity and its consequences (23-25).

The eCBome mediators derived from, or modulated by, gut bacteria may play a role in obesity, dysmetabolism, and intestinal function as previously proposed (1). If we assume that the levels of eCBome mediators in feces are primarily influenced by microbial activity, it can be inferred that a gut microbiota with a decreased relative abundance of families like Christensenellaceae, Clostridiales Family XIII, Rikenellaceae, and Streptococcaceae, along with an increased relative abundance of Burkholderiaceae, may favor the production over the breakdown of NAEs and 2-MAGs within the gut lumen. In the case of Burkholderiaceae, this could lead to enhanced overall fecal microbiota–eCBome signaling through 2-OG, 2-LG, AEA, and DHEA at receptors associated with protective functions, such as GPR55, GPR119, TRPV1, PPARα, and PPARɣ. Whereas, in the case of Christensenellaceae, their lower abundance could lead to higher predominance of signaling by AEA or 2-AG at CB_1_ receptors playing an exacerbating role in dysmetabolism, thus possibly explaining, in part, why these 2 families are negatively associated with obesity. Nevertheless, it is worth considering the possibility that the fecal eCBome mediator influences the proliferation of these bacterial taxa. Given our current understanding, it would be premature to exclude any hypothesis, including the potential for a bidirectional relationship. Other studies have highlighted the association between gut microbiota composition and fecal eCBome mediators. Fornelos et al showed that feces from individuals with intestinal bowel disease contain higher levels of fecal NAEs, partly as a potential consequence of the predominance or reduction of gut microbial species that regulate their metabolism or cellular transport, and that in turn these NAEs produce microbiota changes in culture that reproduce those observed during intestinal bowel disease in both humans and mice (9). Thus, the association between disease or gut microbiota composition and the fecal eCBome suggests a potential contribution to disease symptoms or progression.

Only 2 studies have reported associations between fecal eCBome mediators and health parameters, body mass index and inflammatory bowel disease, respectively (9, 26). Here we report that higher fecal 2-AG, PEA, LEA, and DHEA levels were associated with higher total and visceral fat mass. Higher fecal 2-AG levels were associated with a deteriorated metabolic profile, while fecal OEA levels were negatively associated with HbA1c. The associations between several metabolic parameters and 2-AG levels in the feces mirror those observed in circulation. These results are not surprising considering that 2-AG is notably associated with adverse inflammatory, metabolic, and gut permeability effects through its preferential activation of the CB_1_ receptor (1, 27). Unlike 2-AG, DHEA is generally associated with beneficial anti-inflammatory and metabolic effects through the activation of PPARα, GPR119, and TRPV1 receptors (28). The positive association of DHEA with adiposity and insulin resistance is thus intriguing and may not be merely attributable to the fact that luminal DHEA levels could be already below the minimum effective concentration to be clinically relevant. We have previously reported that circulating OEA levels were positively associated with adiposity measures (eg, fat mass) (3, 12), while we report here that fecal OEA is not associated with adiposity. These results suggest that, unlike other NEAs, fecal and circulating levels of OEA seems to diverge and be associated differently with adiposity and metabolic measures. Further assessment of eCBome mediators in the feces of subjects with obesity and cardiometabolic disease such as type 2 diabetes or dyslipidemia will be needed to address all the above hypotheses. Yet, it is interesting to note how a recent study showed that the administration of *Akkermansia muciniphila* to obese subjects increases the plasmatic levels of 2-PG, an eCBome PPAR-α agonist, while producing beneficial effects on dysmetabolism (29).

Dietary intakes as macronutrients, food groups and patterns are known to shape the gut microbiota composition and activity, but also determine the availability of precursor nutrients for metabolite synthesis. As mentioned above, arachidonic, oleic, and omega-3–derived eCBome mediators were associated with intakes of their corresponding fatty acids in the circulation (3, 30), while only levels of 2-OG and 2-LG show such direct association in the feces. We have previously reported the importance of food patterns in determining the circulating eCBome (3, 21). Similarly, several food groups known as key drivers of the gut microbiota composition (ie, fruits, nuts and legumes, animal proteins) were found here to be associated with NAE and 2-MAG levels in the feces. Moreover, a high-quality dietary intervention provokes changes in the fecal eCBome that, unlike reported previously for the circulating eCBome, appear to be partly independent of their respective fatty acid intakes.

In sum, we can infer that the eCBome mediators we measured in feces may be synthesized or metabolized by the microbial communities or released from epithelial cells being shed into the gut lumen, or both. Previous research highlighted the production of eCBome mediators by gut bacteria (31). Moreover, Baxter et al found that LEA, PEA, and OEA levels are higher in colonoscopy samples from the ascending colon than from the descending colon (26). As gut microbiota composition and metabolic activity differ along the intestinal segments, it would not be surprising to observe site-specific difference in luminal eCBome tone if gut bacteria contributed to NAEs and 2-MAG metabolism. We must emphasize, however, that our results reflect eCBome signaling in feces, and we have currently no data to determine whether our observations can be generalized to the proximal or the distal segment of the colon. Nevertheless, our results support the concept of the contribution of the gut microbiota to the production or catabolism of fecal eCBome mediators. Cytokines, metabolic proteins, and other metabolites are already known to be released through intestinal epithelium shedding in health and in diseases (32). If fecal eCBome mediators originated uniquely from this phenomenon, we would expect that the eCBome profile in the feces, and its response to the diet, reflected those of the host, which seems to be true only in part from our results. Changes in either the intestinal or the circulating eCBome mediator profile in response to a dietary intervention are also not always similar in animal models, which reinforces the present observation (15, 33). Additionally, we have recently shown that levels of NAEs and 2-MAGs in ileal biopsies from individuals with type 2 diabetes do not reflect—and, in fact, in the case of 2-MAGs, are inversely correlated with—the corresponding circulating levels (34). In contrast to molecules that can only be synthesized by host cells, the exact respective contributions of intestinal epithelial shedding and gut microbiota to the fecal eCBome is challenging to assess precisely in humans.

In conclusion, our results seem to support the contribution of the gut microbiota to the production or the catabolism of fecal eCBome mediators. We show that the fecal profile of eCBome mediators is associated with dietary patterns, gut microbiota diversity, and the relative abundance of bacterial families. Considering the crucial role of eCBome mediators in host homeostasis, notably in metabolic and inflammatory functions of the intestinal epithelium, our results should foster further studies aimed at clarifying the link between diet, gut microbiota, and the host to fully understand the role of the fecal eCBome in health and disease.

## Data Availability

Original data generated and analyzed during this study are included in this published article and in the data repositories (NCBI GenBank BioProject ID PRJNA644138 and SRA accession number SUB7687442).

## Clinical Trial Information

NCT03463304 (2018-02-13) and NCT03783260 (2019-01-07)

## Abbreviations

2-AG: 2-arachidonoyl-glycerol
2-DHG: 2-docosahexaenoyl-glycerol
2-DPG: 2-docosapentaenoyl-glycerol
2-EPG: 2-eicosapentaenoyl-glycerol
2-LG: 2-linoleoyl-glycerol
2-MAG: 2-monoacyl-glycerol
2-OG: 2-oleoyl-glycerol
2-PG: 2- palmitoyl-glycerol
AEA: *N*-arachidonoyl-ethanolamine
DHEA: *N*-docosahexaenoyl-ethanolamine
eCBome: endocannabinoidome
EPEA: *N*-eicosapentaenoyl-ethanolamine
LEA: *N*-linoleoyl-ethanolamine
LOD: limit of detection
MFA: multiple factor analysis
NAE: *N*-acyl-ethanolamine
OEA: *N*-oleoyl-ethanolamine
PEA: *N*-palmitoyl-ethanolamine
PPAR: peroxisome proliferator-activated receptor
PUFA: polyunsaturated fatty acid
TRPV1: transient receptor potential vanilloid type-1
